# CalciMAP: High-resolution images showing calbindin- and parvalbumin-expressing neurons across the developing and adult mouse brain

**DOI:** 10.1038/s41597-026-07614-4

**Published:** 2026-06-17

**Authors:** Ingvild E. Bjerke, Olga B. Rogulina, Monika I. Overdevest, Anna Maria Begkai, Arthur Laja, Sharon C. Yates, Heidi Kleven, Ingrid Reiten, Menno P. Witter, Trygve B. Leergaard

**Affiliations:** 1 Neural Systems Laboratory, Institute of Basic Medical Sciences, Department of Molecular Medicine, University of Oslo, Oslo, Norway; 2 Kavli Institute for Systems Neuroscience, NTNU Norwegian University of Science and Technology, Trondheim, Norway

## Abstract

Calcium-binding proteins are abundant in neurons across the brain, serving as essential regulators of neuronal excitability. Dysfunctional regulation of calcium is implicated in a range of neuropsychiatric and neurodevelopmental disorders. Two highly abundant calcium-binding proteins are calbindin and parvalbumin. Despite their importance in brain development, little is known about when and where neurons expressing calbindin and parvalbumin emerge in different brain regions. Most studies into the development of these neurons or their involvement in atypical brain development are limited to one or a few brain regions, and data from these studies are rarely made available. The scarcity of brain-wide information hampers efforts to understand cellular composition changes occurring in the developing brain. Amending this, we here present a brain-wide histological image collection showing calbindin- and parvalbumin-expressing neurons in the mouse brain across five age groups: postnatal day 9, 14, 21, 35 and adult. All the images have been spatially registered to age-specific brain atlases and are made available for both interactive online viewing and download. This collection provides a comprehensive overview of the development of neurons expressing calcium binding proteins in the mouse brain and is tailored for use in analytical workflows to quantify changes across development.

## Background & Summary

Calcium signalling is essential for normal brain function, with transient fluctuations in intracellular levels affecting synaptic plasticity, neurotransmitter release, and gene expression^1,2^. Intracellular calcium concentrations must be tightly regulated, and dysregulation of calcium signalling has been implicated in a range of disorders such as Alzheimer’s disease, schizophrenia, and bipolar disorder^3,4^. In the healthy brain, an extensive “calcium signaling toolkit” of regulatory proteins contributes to tuning the spatial and temporal dynamics of intracellular calcium^5^. As important components of this regulatory system, calcium-binding proteins are located in the cytosol, ready to bind and efficiently buffer excess intracellular calcium^6,7^.

Two highly abundant calcium-binding proteins in the brain are parvalbumin and calbindin (also referred to as calbindin1 or calbindin-D28K), and neurons expressing these proteins are widely distributed across the brain^8–10^. Parvalbumin- and calbindin-positive neurons are involved in a range of functions related to spatial navigation^11,12^, memory processes^13–15^ and social behaviour^16^. We have previously quantified calbindin and parvalbumin positive neurons across the adult mouse brain^8^, showing that these neurons have a largely complementary distribution across the brain that fits well with their respective functional roles^11,16^. However, calbindin- and parvalbumin-expressing neurons also have important roles in the developing mammalian brain, where they contribute to various aspects of maturation and establishment of different brain systems^17^. Changes in calbindin and parvalbumin levels have been associated with altered sensory^18,19^ and motor system^20^ development, as well as neurodevelopmental and mental disorders, including psychiatric fetal hydrocephalus^21^, schizophrenia^22,23^, autism^24^, and motor neuron disease^25^. To understand how parvalbumin and calbindin expressing cells establish their functional roles, detailed information about their regional and subregional distribution across the whole brain during development is required. Such data are scarcely available. Some studies have investigated the expression of calbindin or parvalbumin through development^26,27^. However, the focus has mainly been on cortical development, with results presented through qualitative observations and example images. Without access to underlying data, it is difficult to compare existing information with new findings.

Several recent efforts have been made to make large collections of immunohistochemically stained sections digitally available and re-usable in accordance with the FAIR principles^28–32^. An important aspect of these new approaches is spatial registration of images to 3D brain atlases to make images comparable and interoperable with other data mapped to the same reference framework^33–35^. Furthermore, the continuous development of user-friendly semi-automated analysis techniques over the last decade^35–37^ ensures that quantitative information can be extracted from such images in an efficient manner. These efforts have often focused on the adult brain, but more recent work has produced reference atlases, tools and large datasets mapping cells across the developing mouse brain^38–40^. Notably, Liwang and colleagues recently mapped microglia and GABAergic neurons in several early postnatal stages using age-specific serial two-photon tomography templates^38^. Utilizing these and other published templates^41^, we created a temporally continuous atlas of postnatal development (DeMBA) and integrated this atlas into tools for spatial registration of histological images^39^. These advancements bring new opportunities for systematic brain-wide quantitative analyses of neural markers during development. In this context, there is broad interest for cellular resolution image data showing the development of various cell types, including those expressing calcium-binding proteins.

We present a collection of high-resolution images from developing male and female mice at five stages (postnatal day 9, 14, 21, 35 and adult) showing the distribution of parvalbumin and calbindin positive cells across the brain. All section images are registered to age-specific versions of the Allen mouse brain Common Coordinate Framework^39,42^, facilitating comparative analysis across age groups. The image collection, referred to as CalciMAP, is shared via the EBRAINS Knowledge Graph, where it is available for download as well as interactive inspection via a web viewer. The images are highly suitable for semi-automated, brain-wide analyses, which could yield important insights into the normal development of calcium-binding protein expression in the mouse brain.

## Methods

### A note on data preparation and acquisition

The CalciMAP project was initiated in 2018, with the aim of collecting brain-wide quantitative data describing calbindin- and parvalbumin-expressing neurons in adult mice. Parvalbumin stained sections were acquired from four brains at the Kavli Institute, Norwegian University of Science (NTNU) and Technology, Norway, while calbindin stained sections from six brains were collected at the Neural Systems Laboratory, University of Oslo (UiO), Norway. These images were analysed and reported in our previous study^8^. Corresponding datasets were shared via the EBRAINS Knowledge Graph, containing parvalbumin data from four adult mice and calbindin data from six adult mice^43–45^. In 2021, the collection was expanded to include brains from developing animals, all acquired at the Neural Systems Laboratory, UiO. At the same time, we acquired more parvalbumin and calbindin stained sections from the adult, using stored brain sections from the same group of animals previously used for the adult calbindin images.

Experimental procedures involving animals were approved by the Norwegian Food Safety Authority and carried out in accordance with national regulations, which follow European Union legislation of the 2010/63/EU directive on the protection of animals used for scientific purposes. Procedures at UiO were approved by the Animal Use and Care Committee at the Institute of Basic Medical Sciences (Faculty of Medicine) and the Norwegian Food Safety Authority (project numbers 12717 and 28114). Procedures at NTNU were approved by the Animal Welfare Committee of NTNU and the Norwegian Food Safety Authority (project numbers 8083 and 8082).

**Fig. 1:**
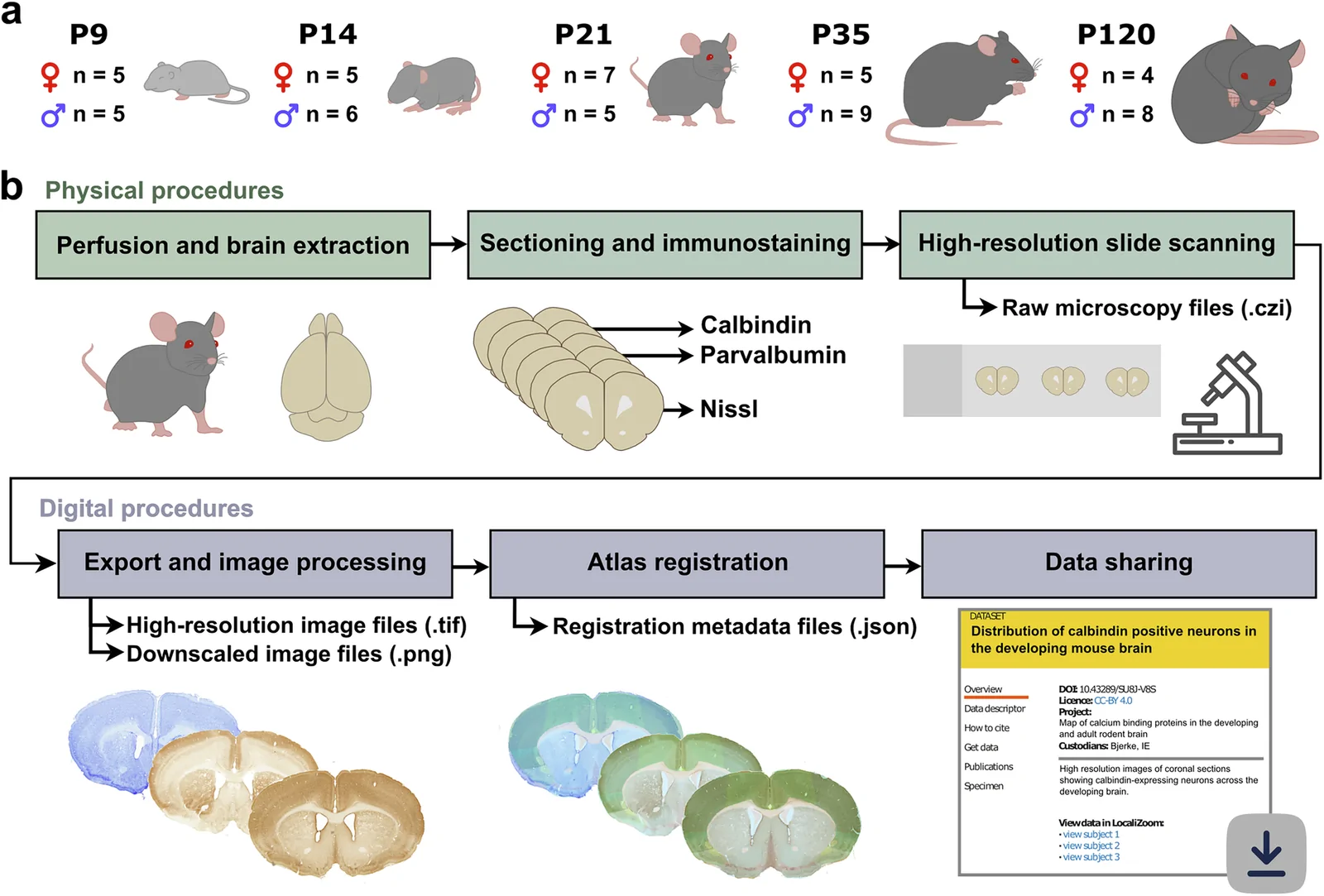
Overview of the CalciMAP image collection, subject groups and procedures. (**a**) Overview of subject groups with sample sizes for male and female mice. (**b**) Data acquisition procedures, broadly categorized into physical processing (green boxes) steps and digital steps (grey boxes) yielding the data files that make up the five datasets shared via the EBRAINS Knowledge Graph.

The same principal methods of paraformaldehyde perfusion fixation, immunohistochemistry, and spatial registration to atlas were used for all animals; importantly, the same primary antibodies for calbindin and parvalbumin were used for all brains. Procedural details concerning the perfusion, sectioning and immunohistochemistry (e.g. buffer solutions or incubation times) differ slightly between brains processed in the NTNU laboratory (four adult mice used for parvalbumin and cresyl violet staining) and the UiO laboratory (all remaining cases). The histotechnical procedures summarized in the sections below are based on the UiO procedure. Methodological details concerning brains processed at NTNU are described in the data descriptor accompanying this dataset shared via the EBRAINS Knowledge Graph^46^. The main points where the NTNU procedure differs from the UiO procedure are (1) use of sodium pentobarbital for anaesthesia; (2) use of 30 µm horizontal sections with 1:4 sampling; (3) export of TIF images at 50% of the full resolution. Figure 1 summarises the subject groups and procedures.

### Animals and perfusion

Male and female C57BL6/J mice of five age groups (postnatal day (P) 9, 14, 21, 35 and adult; Fig. 1a) were used in this study. Adult animals were 4–6 months old. Adult and P35 mice were ordered from Janvier labs and acclimatized for a minimum of ten days before perfusion, while the younger animals were bred in-house. Prior to perfusion, all mice were given a unique numerical ID, hereafter referred to as the “mouse ID”. All perfusions were performed between 8 AM and 12 noon at the day of each specific age (for P21 and P35, within a range of +/− 1 day). Mice were weighed and given a lethal dose of ZRF-cocktail (0.1 ml/10 g) by intraperitoneal injection. Note that mice in the adult group were anaesthetized with isoflurane prior to this injection. Upon absence of any reflexes as measured by toe-pinch, mice were transcardially perfused with ice-cold 2% dextran followed by 4% paraformaldehyde. The perfusion with paraformaldehyde was continued for 8–14 minutes depending on the age, with less time required in the young mice, until the body was rigid. The quality of the perfusion was assessed by the paleness of the liver and stiffness of the body, and this assessment was noted to keep a record of perfusion quality for each brain (see Supplementary File 1). After perfusion, the brains were dissected and stored in 4% paraformaldehyde for 12–24 hours. The next day, they were transferred to a solution of 0.1% PFA for storage or directly cryoprotected.

### Sectioning

Prior to sectioning, the brains were cryoprotected in a series of sucrose solutions at ascending concentrations (10%, 20%, and 30% sucrose, each time until they sank). A small incision was made in the right hemisphere of the brain to ensure that left-right orientation could be determined later. Brains were cut coronally into two blocks to have a stable surface for sectioning of each block. As this led to some tissue loss at the end of each block, the brains were cut at different anteroposterior levels to avoid losing the same regions each time. Brains were sectioned coronally at 40 µm using a freezing sliding microtome (cutting temperature −20 °C) and stored at 4 °C in 0.1% PFA in NAPI until further processing.

### Immunohistochemistry

Every sixth section across the whole brain was used for immunohistochemistry, from the tip of the olfactory bulb to the end of the cerebellum. A step-by-step version of the protocol described below is available from protocols.io^47^. Unless otherwise specified, steps were performed at room temperature. On the first day, sections were washed with phosphate buffered saline (PBS, pH 7.4) and subsequently blocked with 3% hydrogen peroxide (H_2_O_2_) for five minutes. After washing again, sections were incubated in a blocking solution (PBS with 10% normal donkey serum, 1% bovine serum albumin, and 0.5% triton) for one hour. Sections were then washed again and incubated in primary antibody overnight at 4 °C on a rotating table. The primary antibodies used were a monoclonal mouse anti-calbindin antibody (RRID:AB_10000347) and a monoclonal rabbit anti-parvalbumin antibody (RRID:AB_2631173). These were diluted to a concentration of 1:5000 (calbindin) and 1:4000 (parvalbumin) in an incubation buffer of PBS with 3% normal donkey serum, 1% bovine serum albumin and 0.1% triton. On the second day, sections were washed in PBS with 0.2% triton, and subsequently incubated with the secondary antibodies. The secondary antibodies used were a sheep anti-mouse antibody (RPN1001V, GE Healthcare) for calbindin and a donkey anti-rabbit (RPN1004V, GE Healthcare) for parvalbumin. These were diluted to a concentration of 1:500 using the same incubation buffer as for the primary antibodies. Sections were then washed again in PBS (first with 0.2% triton and then with PBS only), before being incubated for 30 minutes in an avidin-biotin solution (ABC kit, pk-6100, Vector Laboratories). Sections were washed again and then incubated with a solution of 3,3-diaminobenzidine (DAB) and H_2_O_2_ (DAB kit, sk-4100, Vector Laboratories). The reaction was stopped by washing sections in sterile water after 5 and 2 minutes for calbindin and parvalbumin staining, respectively. For parvalbumin staining, the DAB solution was kept on ice until incubation to slow the reaction time. Sections were washed in sterile water two times, and then transferred to PBS until mounting.

Sections were mounted on gelatine coated slides, with 3-4 sections mounted on each slide and the midline of the section mounted perpendicularly to the long axis of the slide. Sections were air dried before being dehydrated in an ascending series of ethanol (2 minutes using 70% ethanol, and 2 × 2 minutes in 96%, and 100% ethanol). Lastly, sections were cleared with 2 × 2 minutes in xylene, coverslipped with Eukitt, and left to dry overnight before scanning.

For all brains, a 1:6 series was used for Nissl staining, which is helpful for interpretation of anatomical features during image organization and spatial registration. Sections were mounted on gelatine-coated slides (typically three or four sections per slide) and dipped briefly in sterile water before being incubated in a cresyl violet-solution for 20 minutes. They were then washed in an ascending series of ethanol solutions before being cleared and coverslipped, as described above.

### Scanning and image organization

Slides were scanned using an Axioscan (Zeiss) slide scanner with a 20 × /0.80NA Plan-Apochromat objective, resulting in high-resolution images with 0.22 × 0.22 µm pixels. The resulting CZI files were exported using Zen 2.6 blue edition (RRID:SCR017183), after adjusting the white balance of each scanned slide. Images were exported at full resolution in tiled TIF format. A detailed protocol for image export and file naming convention has been published at protocols.io^48^.

The images (with one image corresponding to one brain section) were organized from anterior to posterior, using a file naming scheme that included the mouse ID, age, sex and stain as a prefix and the brain section number as a suffix. Sections were named using their absolute index in the brain they came from, thus reflecting their 1:6 sampling and preserving information about the relationship of sections (calbindin, parvalbumin and Nissl stained) across series. All images were manually inspected to ensure optimal consistency of section orientation. First, we determined whether a section had been flipped or heavily rotated during mounting. Such images were adjusted using Nutil Transform^49^ (RRID:SCR017183, v0.6.0 - v0.8.0). Secondly, we looked for instances where parts of the section (in regions of the brain where several dissociated tissue pieces make up the section) had been flipped during mounting. For example, towards the back of the brain, the brainstem may be flipped even if the cortical hemispheres are not. In such cases, we manually adjusted individual parts of the sections using Adobe Photoshop (RRID:SCR_014199). We also adjusted sections where individual parts had been mounted with excess distance between them, as it can be difficult or impossible to achieve a good spatial registration of such images to the brain atlas (see section below). To speed up the Photoshop processing, we used the actions function to perform adjustments on low-resolution images (requiring significantly less time to open and manipulate) and used a custom Python script to allow such actions to be applied on batches of the high-resolution images. Protocols describing how to assess image order, flipping, and mounting errors, as well as adjusting the latter cases in Photoshop, have been published on protocols.io^50,51^ and scripts to facilitate these processes are available from GitHub (see Code Availability for details). Image pre-processing steps (including rotation, flipping and Photoshop adjustments) have been documented for every section series, and tabular overviews of this are available as metadata with the published datasets.

### Registration of images to reference brain atlas

All images were spatially registered to age-specific reference brain atlas versions from the Developmental Mouse Brain Atlas (DeMBA)^39,52^. The DeMBA atlas was created using templates shared by Liwang and colleagues and by Newmaster and colleagues^38,41^, and interpolated to cover all postnatal days between 4 and 56. We used these templates and corresponding segmentations derived from the Allen mouse brain Common Coordinate Framework (Allen CCF)^42^. This allowed us to use an exactly age-matching atlas for each developmental age group while preserving comparability between them.

To perform a linear registration of each brain section series to the relevant atlas, we used the QuickNII software (RRID:SCR_016854)^33^. This software allows the user to create custom-defined cutting planes through a brain atlas, making it possible to account for slight deviations from the standard plane. For each section series, such deviations were determined by inspecting anatomical landmarks^53^ across hemispheres and in dorsal and ventral parts of the section. The anteroposterior positions of sections with particularly prominent landmarks (typically including the anterior striatum, merging of the anterior commissure, anterior hippocampus, the superior colliculus, and the inferior olive)^53^ were determined first, and the remaining section positions, estimated by the propagation in the software, were only minimally adjusted. Calbindin-, parvalbumin- and Nissl-stained sections from the same brains were registered at the same time and within the same file to ensure that the registration would accurately represent the relative positions of the sections across series. The registration of each section was non-linearly refined using VisuAlign (RRID:SCR_017978)^54^. The QuickNII and VisuAlign versions used here are bundled with each of the age-specific brain atlases and are available through the EBRAINS Knowledge Graph^52^.

### Analysis of variability in staining intensity

To assess the variability of staining intensity in the material, we analysed all P21 calbindin- and parvalbumin-stained sections using the QUINT workflow, which is tailored for atlas-based quantification of features in images^35^. Calbindin- and parvalbumin-positive cell bodies were segmented with the cellular segmentation algorithm Cellpose 4.0.4^55,56^, training our own deep-learning models based on the Cellpose Segment Anything Model (CPSAM)^55^. Separate models were trained for calbindin and parvalbumin datasets, using 256 × 256 pixel chunk images from different subjects and brain regions. Each model was trained on images from two animals and validated on images from two separate animals, using chunk images from a variety of regions for both training and validation. Standards performance metrics were calculated for both models, yielding overall F1 scores of 0.94 for parvalbumin (precision = 0.97, recall = 0.90, n = 645 ground truth cells across 52 images) and 0.92 for calbindin (precision = 0.91, recall 0.93, n = 1720 ground truth cells across 47 images). The models were applied to downscaled (20% of full resolution images) images. Cellpose model training, validation and application was achieved using python scripts available from https://github.com/ingvildeb/cellpose_scripts. Segmented images were binarized prior to quantification of cells per atlas region, using the Neuroimaging Utilities tool Nutil v1.0.5^49^.

Three regions were selected for analysis, with data pooled from relevant subregions in the reference atlas region reports produced by Nutil: the basolateral nucleus of the amygdala (including anterior, posterior and ventral parts), the anterior cingulate cortex (including all layers of the dorsal and ventral parts), and the caudoputamen. For each region, density distribution was assessed across sections to flag potential damaged sections. The density in each region and section was compared to a rolling local median, calculated across neighbouring sections (window size = 3). Sections were flagged if the density showed a decrease or increase relative to these adjacent sections. All flagged sections were manually reviewed, and those affected by artifacts or damage were excluded from the validated dataset. For each brain, validated section-level counts were aggregated across sections to arrive at region-wise total counts and densities. Densities were expressed as cells per square micron. To evaluate investigator-associated staining variability, densities were compared across investigators separately for each marker and region, using Kruskal-Wallis tests, followed by Benjamini-Hochberg correction for multiple comparisons. All the raw data from the technical variability assessment are made available with the relevant CalciMAP datasets^57,58^, and all the code used to summarize the findings are available from Github (see Code availability).

## Data Records

The CalciMAP image collection includes parvalbumin-, calbindin- and Nissl- stained sections from a total of 63 male and female mice across five age groups (see Table 1 for a breakdown of the number of males and females per age and stain). All the CalciMAP data are shared through five datasets available via the EBRAINS Knowledge Graph^46,57–60^.

**Table 1:** Overview of the CalciMAP image collection.

Staining	Age	Sex	# of subjects	Average # of images per subject	Total # of images
Calbindin	P9	F	5	41	204
		M	5	43	213
	P14	F	4	50	201
		M	6	45	267
	P21	F	7	50	351
		M	5	46	232
	P35	F	5	55	275
		M	7	52	363
	Adult	F	4	47	189
		M	4	49	197
Parvalbumin	P9	F	5	41	206
		M	5	42	208
	P14	F	5	46	232
		M	6	44	263
	P21	F	7	49	344
		M	5	47	234
	P35	F	5	55	273
		M	7	51	359
	Adult	F	4	47	189
		M	5	45	223
Nissl	P9	F	5	37	184
		M	5	38	192
	P14	F	5	43	215
		M	6	44	266
	P21	F	7	47	326
		M	5	47	233
	P35	F	5	52	261
		M	9	52	469
	Adult	F	4	51	202
		M	8	47	379
			**Total:** 63	**Average # of images across subjects:** 47	**Total:** 7750

The table summarizes the number of subjects, average number of images, and total number of images for each stain, age group and sex. Because the subjects used for the different stains are largely overlapping, most subject are represented multiple times in the column “# of subjects”; the total number of subjects is 63.

Within each dataset, images are organized in folders according to age and mouse ID. For each subject, the raw files (CZI format) and final processed image (TIF format) files are shared in separate folders. Each CZI file represents a slide with 3-4 brain sections. Downscaled thumbnail versions (PNG format, 20% and 2% of original resolution) of the images are also available. The 20% size thumbnails provide a good compromise between file size and resolution and can be used for semi-automated analyses (see the Use Case section for an example). The 2% size thumbnails were used for spatial registration, and the nonlinear registration file (JSON format) is provided in the corresponding folder for each subject. Table 1 provides an overview of the number of subjects and images for each stain and age group.

## Technical Validation

### Antibody specificity

To ensure the antibodies were producing specific staining, we included 1-2 negative control sections in each experiment. These sections underwent the entire immunohistochemical procedure described in the methods, except for the omission of the primary antibody incubation. The control sections are not included in the TIF image series but are available in the CZI files (any slide that contains a control section has the suffix “_control” in the file name). The negative control sections consistently showed absence of any specific staining, only showing faint background staining. In some cases of poor perfusion quality, we did see staining of blood vessels in the negative controls for the calbindin antibody (see section on “Perfusion quality” below for details).

### Perfusion quality

The quality of a perfusion can influence results of immunohistochemical staining. A poorly perfused brain may be soft and more difficult to dissect, yielding higher risk of tissue damage; also, any remaining blood cells may react with the DAB and create more background staining than in a well-perfused brain. While most of the perfusions in this study were of high quality, a few were less successful. As we generally obtained acceptable results with immunohistochemistry for these brains, we still included them in the dataset and have included an overview of the perfusion quality for each mouse in Supplementary file 1. We noticed that calbindin stained sections from suboptimally perfused mice showed some unspecific staining of blood vessels. Stained blood vessels were also observed in negative control sections of these animals, but only in the calbindin stained material. Given that the only difference between these sections and the parvalbumin control sections is the secondary antibody, this may be caused by the secondary antibody reacting with endogenous proteins in blood cells. For the parvalbumin stained material, the immunohistochemical staining was not noticeably different depending on the quality of the perfusion, although some of the animals with poor perfusion also showed a higher level of tissue damage.

### Variability in staining intensity

Despite the use of a standardized and highly detailed protocol, we observed variability in staining intensity across sections and series. Many subtle factors may affect this, and one potential source of this variability was that immunohistochemical experiments were performed by six different investigators. To minimize the effect of this on future analyses, we ensured that no investigator systematically processed all brains from a group and most investigators processed brains across multiple groups. To understand how staining variability might affect downstream quantitative analyses, we analyzed all the data from the postnatal day 21 group. We used the QUINT workflow for atlas-based quantification of cells in images from each brain. Calbindin and parvalbumin stained cell bodies were extracted using the deep-learning based software Cellpose 4.0 and the segmented images were combined with the non-linear atlas registration data presented here using Nutil.

When considering technical variability, it is essential to also consider the biological variability. We focused on three brain regions with distinct staining characteristics: the basolateral amygdala, the caudoputamen and the anterior cingulate cortex. These areas together represent a broad range of the variability in appearance of calbindin and parvalbumin stained cell bodies observed across the CalciMAP collection. Parvalbumin staining generally shows dark, well-defined cell bodies that are easy to distinguish from background, whereas calbindin staining is more heterogeneous. In cortical and striatal areas, calbindin cells are often weakly stained and densely packed, making them difficult to distinguish from background. The amygdala, on the other hand, shows larger, darker and more distinct calbindin stained cells.

**Fig. 2:**
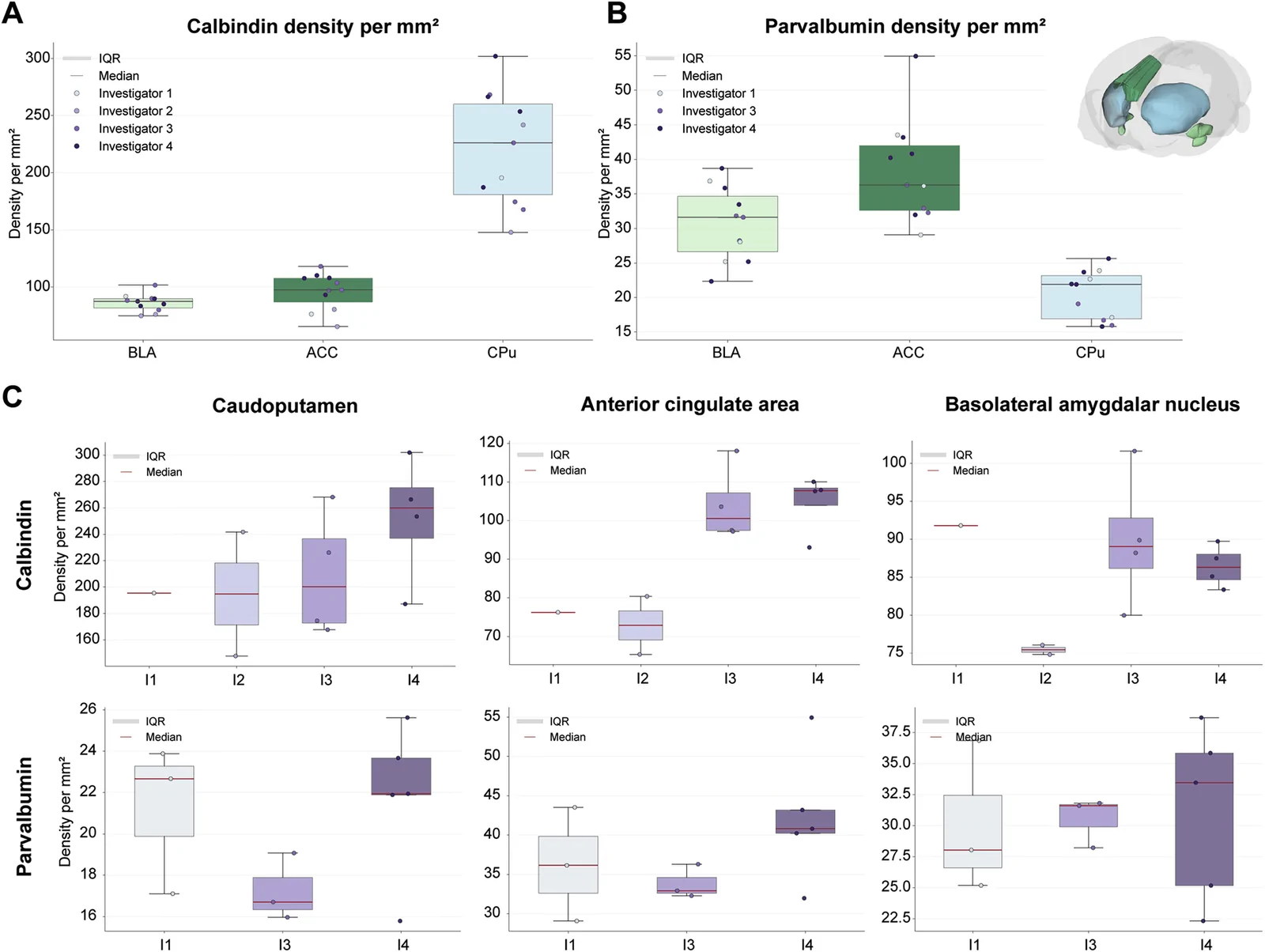
Assessment of variability associated with investigator performing immunohistochemistry. Cell density estimates were obtained from P21 calbindin and parvalbumin datasets stained by four different investigators. (**A,B**) Cell density estimates from three representative regions for calbindin (**A**) and parvalbumin (**B**) obtained by using the QUINT workflow. Each point represents one brain and is color-coded by the investigator who performed the immunohistochemistry. Boxplots show the interquartile range (IQR) and median. (**C**) Investigator-wise distributions of estimated cell densities for each region, shown separately for calbindin (top row) and parvalbumin (bottom row). Abbreviations: ACC, anterior cingulate area; BLA, basolateral amygdalar nucleus; CPu, caudoputamen; I, investigator.

We quantified cell densities per square millimeter and observed variations across brain regions as expected from qualitative observations. For example, we saw a high calbindin density and a relatively sparse parvalbumin population in the caudoputamen (Fig. 2A,B). Comparison of estimated cell densities by the investigator who performed immunohistochemistry revealed some variation (Fig. 2C). Calbindin cell densities were more variable across investigators in cortical and striatal regions than parvalbumin cell densities. This is consistent with a more heterogeneous staining profile of calbindin cells, which most likely amplifies the impact of variation in staining intensity (Fig. 2C). However, no statistically significant investigator effect was detected in any region for either marker (Kruskal-Wallis tests, all FDR-adjusted p values > 0.33; Fig. 2C). Taken together, this analysis suggests that while staining variability is present in the dataset, it does not appear to be systematically driven by which investigator performed the immunohistochemistry. Nevertheless, variability in staining and tissue quality is present and should be considered for quantitative analyses. We recommend that users evaluate their regions of interest for missing tissue, suboptimal staining quality, or other local artifacts, and account for this when aggregating results. All the data underlying the variability analysis have been deposited with the relevant datasets on EBRAINS^57,58^, and all the code and metadata used for analysis is available from the CalciMAP Github repository (see Code Availability).

### Registration quality

The registration of atlas to the brain section images with the software QuickNII and VisuAlign is a flexible, user-guided process involving manual adjustments based on neuroanatomical expertise^54^. Therefore, conventional quantitative evaluation metrics (e.g. landmark-based registration error) are not applicable at scale and may not provide independent validation when performed by the same researcher. To ensure accurate and consistent registration results, all registrations were either performed or validated by an experienced neuroanatomist. To ensure transparency and reproducibility, all registrations are available for interactive inspection through the viewer links on the dataset landing pages. The registration files are also available for download, allowing users to directly assess, reproduce or refine registrations as needed.

## Data Overview

The image collection presented in this paper is available via the EBRAINS Knowledge Graph, as five datasets shared under a CC-BY licence. The landing page of each dataset contains information about the subjects, and links to download individual files or the whole dataset. We provide both the raw microscopy files (CZI format) and processed image files (TIF format) optimized for further analysis. We also share low-resolution image versions (PNG format) used for spatial registration, as well as registration files (JSON format) specifying spatial registration parameters for each image.

**Fig. 3:**
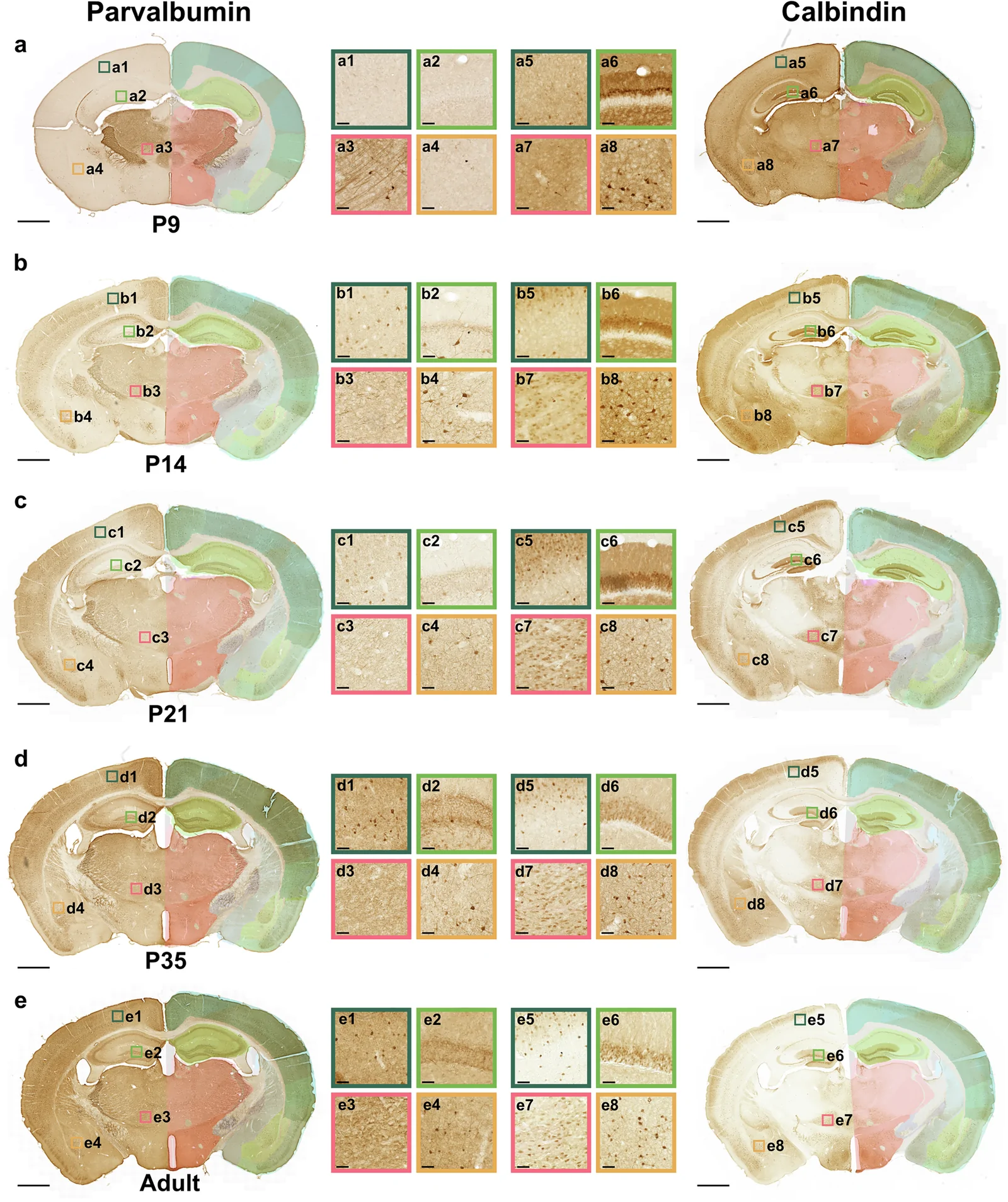
Comparing the development of calbindin and parvalbumin expression. The CalciMAP collection is available for online, interactive viewing with custom-cut atlas maps, facilitating comparison of ages and regions of interest. The figure shows an example coronal section for each stain and age, with a transparent view of each corresponding atlas plate shown on the right hemisphere. Four regions are highlighted with magnified images in the middle panel (cortex, a1-e1 and a5-e5; hippocampus, a2-e2 and a6-e6; thalamus, a3-e3 and a7-e7; amygdala, a4-e4 and a8-e8). Scale bars are 1 mm (a–e) and 50 µm (insets).

The landing page also includes links to an online viewer for each subject, allowing the images to be viewed and explored online. Visual inspection and comparison of data across age groups is facilitated by the customized and non-linearly refined atlas maps (Fig. 3), through which corresponding regions of different brains and age groups can be identified and inspected. Via inspection of sections in the online viewers, several striking features of calbindin and parvalbumin development can be noted (Fig. 3). For example, parvalbumin-expressing cells are largely absent from the cortex and hippocampus at P9 (Figure 3a1-2), whereas the thalamus shows abundant staining, mainly of fibres (Figure 3a3). Calbindin-expressing cells, on the other hand, are abundantly expressed in certain hippocampal layers (Figure 3a6), and some scattered cells can be seen in the cortex already at this age (Figure 3a5). In contrast, there are few calbindin-expressing cells at P9 in the thalamus (Figure 3a7). Example images from all ages with focus on a few selected areas are shown in Fig. 3, but all the images from all ages can be explored online at full resolution.

## Usage Notes

**Fig. 4:**
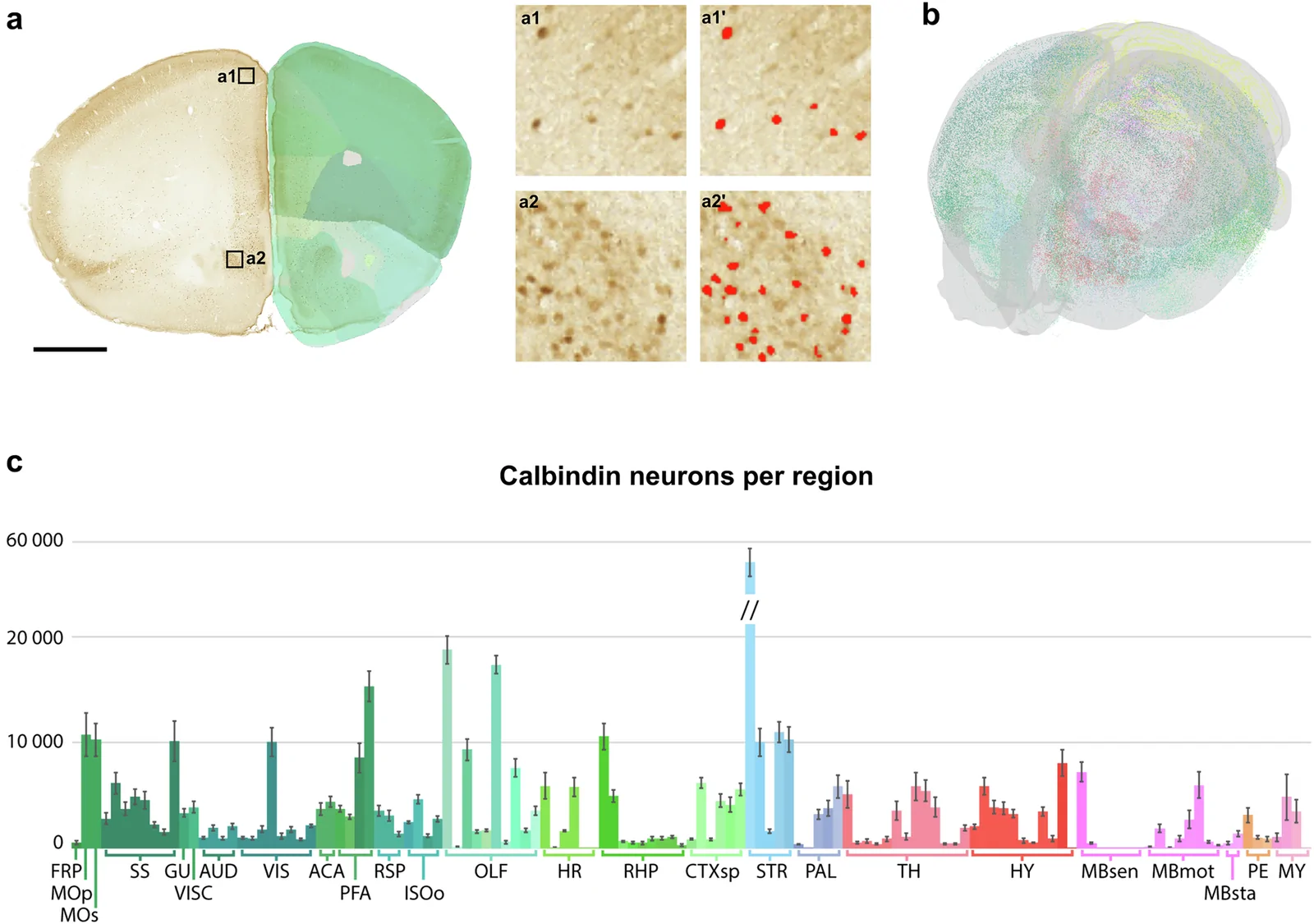
Semi-automated analysis of CalciMAP data. (**a**) Calbindin-stained coronal section from one of the CalciMAP subjects (mouse6, adult group). With the corresponding custom-cut atlas map shown on the right side. Insets show magnified views from the anterior cingulate cortex (a1) and the anterior olfactory nucleus (a2) with corresponding segmentations made using ilastik (a1’-a2’). (**b**) Point cloud visualization of all objects across the entire brain, with points colour coded according to the regions of the Allen mouse brain CCF. (**c**) Quantification of calbindin neurons per region (graph adapted from Bjerke *et al*.^8^), with each bar representing a region in the Allen mouse brain CCF.

CalciMAP is a curated image collection where mounting errors have been corrected, and every image is non-linearly registered to age-specific brain atlases. The images can readily be explored using the web-microscopy tool LocaliZoom^54^ via links available from the EBRAINS data cards. This viewer tool allows users to interactively explore serial high-resolution images online, guided by atlas overlay images providing anatomical reference facilitating qualitative evaluation and comparison between regions and with other data registered to the same reference atlas. See earlier work^31^ for examples of qualitative and semiquantitative evaluation of histological labelling using the LocaliZoom viewer tool. The atlas-registered images are also well suited for semi-automated analysis, for example using the QUINT workflow^35^. This workflow combines segmented images with the non-linear atlas registration metadata resulting from QuickNII and VisuAlign, allowing quantification and visualization of segmented objects. Our publication reporting numbers and densities of calbindin and parvalbumin in adult mice provides an example of such an analysis^8^. Briefly, the user would segment images, e.g. using the ilastik software^37^ or Cellpose^55^ and combine the results with the atlas registration metadata provided for the images (Fig. 4a) using the Nutil software^49^. This allows for visualization (Fig. 4b) and quantification (Fig. 4c) of features of interest. Figure 4 shows an example of such an analysis for calbindin stained material from one of the adult subjects (segmentations and quantitative results from our previous publication^8^). As the developmental brain atlas used for spatial registration (DeMBA^39^) is fully integrated in the QUINT workflow, similar analysis can be performed across all ages (for an example of such an analysis, see the publication presenting DeMBA^39^).

The CalciMAP collection compliments existing resources mapping cell types in the developing mouse brain^38,41^. Notable previous studies mapping cellular features across developing brains have focused on oxytocin receptor positive cells^41^, GABA-ergic cells and microglia^38^, dopamine receptor positive cells^32,61^, and vasculature^62^. CalciMAP contributes valuable insight into calcium-binding protein expressing cells, for which previous studies have been qualitative and limited to cortical regions^27^. Several of the previous studies have also produced age-specific atlas templates to facilitate their mapping efforts. This has created a diverse landscape of developmental atlases built with different imaging modalities, e.g. magnetic resonance imaging^40^, serial two-photon tomography (STPT)^38,39,41^ or light sheet fluorescence microscopy^62^. The atlas used here, DeMBA, was built on STPT templates and integrated in tools for spatial registration of histological images^39,54^, the latter which makes it particularly suitable for use with the CalciMAP data. Most of the recently published atlases for developing brains make use of the Allen CCFv3 segmentations, making data generated using the different atlases comparable at the level of the brain region terminology. Future work should focus on making all these diverse resources interoperable on a spatial level, facilitating co-visualization and deeper integration of data obtained in the growing body of research on cell type development.

## Supplementary information

Supplementary information

## Data Availability

The datasets are available at EBRAINS: Distribution of calbindin-positive neurons in the normal adult mouse brain (v3). [https://doi.org/10.25493/J4S8-A84]; Distribution of calbindin-positive neurons in the developing mouse brain. [https://doi.org/10.25493/ND61-ZCJ]; Distribution of parvalbumin-positive interneurons in the normal adult mouse brain (v3). 2026. [https://doi.org/10.25493/PF8V-YMU]; Distribution of parvalbumin-positive neurons in the developing mouse brain (v3). [https://doi.org/10.25493/F3F9-Z11]; Brain-wide Nissl datasets from developing and adult mouse brains (v2). [https://doi.org/10.25493/CWCF-YYY].

## Code availability

Code associated with this study is available in two related GitHub repositories. Reusable functions for processing serial brain section images are available from https://github.com/ingvildeb/brain_section_scripts. Project-specific scripts used to implement the workflow for the data presented here are available from https://github.com/ingvildeb/CalciMAP. The latter repository reflects the organization of a specific project environment and is provided mainly for documentation and reproducibility purposes rather than for direct re-use.
